# Fine mapping and characterization of *RLL6* locus required for anti-silencing of a transgene and DNA demethylation in *Arabidopsis* *thaliana*

**DOI:** 10.3389/fgene.2022.1008700

**Published:** 2022-09-26

**Authors:** Xiangyu Wang, Min Wang, Jie Dai, Qianqian Wang, Honggui La

**Affiliations:** College of Life Sciences, Nanjing Agricultural University, Nanjing, Jiangsu, China

**Keywords:** arabidopsis thaliana, reduced LUC luminescence 6 (RLL6), DNA demethylation, map-based cloning, whole-genome bisulfite sequencing (WGBS), repressor of silencing 1 (ROS1)

## Abstract

DNA methylation patterns in plants are dynamically shaped by the antagonistic actions of DNA methylation and demethylation pathways. Although the DNA methylation pathway has been well studied, the DNA demethylation pathway, however, are not fully understood so far. To gain deeper insights into the mechanisms of DNA demethylation pathway, we conducted a genetic screening for proteins that were involved in preventing epigenetic gene silencing, and then the ones, which were also implicated in DNA demethylation pathway, were used for further studies. Eventually, a mutant with low luciferase luminescence (low LUC luminescence) was recovered, and named *reduced LUC luminescence 6–1* (*rll6-1*). Map-based cloning revealed that *rll6-1* mutation was located on chromosome 4, and there were a total of 10 candidate genes residing within such a region. Analyses of genome-wide methylation patterns of *rll6-1* mutant showed that mutation of *RLL6* locus led to 3,863 hyper-DMRs (DMRs for differentially methylated regions) throughout five *Arabidopsis* chromosomes, and elevated DNA methylation level of *2 × 35S* promoter, which was similar to that found in the *ros1* (*repressor of silencing 1*) mutant. Further analysis demonstrated that there were 1,456 common hyper-DMRs shared by *rll6-1* and *ros1-7* mutants, suggesting that both proteins acted together in a synergistic manner to remove DNA methylation. Further investigations demonstrated that mutation of *RLL6* locus did not affect the expression of the four genes of the DNA glycosylase/lyase family. Thus, our results demonstrate that *RLL6* locus-encoded protein not only participates in transcriptional anti-silencing of a transgene, but is also involved in DNA demethylation pathway.

## Introduction

DNA methylation is a conserved epigenetic mark that plays important roles in plant and vertebrate development, genome stability, and gene regulation (Law and Jacobsen, 2010). In animals, 5-methylcytisines (5-meCs) predominantly occur at CG dinucleotides, whereas in plants 5-meCs are found in CG, CHG and CHH contexts (where H is A, C or T) (He et al., 2011). RNA-directed DNA methylation (RdDM) is able to establish the *de novo* DNA methylation in all sequence contexts (Matzke and Mosher, 2014). Methylation in each context is mainly maintained by three types of DNA methyltransferases: the CG and CHG methylation is maintained by methyltransferase 1 (MET1) and chromomethylase 3 (CMT3), respectively, while the CHH methylation is maintained by both DOMAINS REARRANGED methylase 2 (DRM2) and chromomethylase 2 (CMT2) (Cao and Jacobsen, 2002; Law and Jacobsen, 2010; Zemach et al., 2013).

However, DNA methylation can be counteracted by DNA demethylation *in vivo*. According to the way it happens, the DNA demethylation can be classified into two categories: passive DNA demethylation and active DNA demethylation (Wu and Zhang, 2010). The former occurs during DNA replication when the activity of a DNA methyltransferase is inhibited (Wu and Zhang, 2010). On the other hand, the active DNA demethylation is catalyzed by DNA demethylases, and has been found to perform prominent functions in preventing the spread of DNA methylation to the neighboring regions (Zhu et al., 2007). In mammals, active DNA demethylation is initiated by TEN-ELEVEN-TRANSLOCATION (TET) enzymes or ACTIVATION INDUCED deaminase/APOLIPOPROTEIN B RNA-EDITING CATALYTIC COMPONENT-1 (AID/APOBEC), followed by the actions of DNA glycosylase THYMINE DNA glycosylase (TDG) or METHYL-CpG-BINDING DOMAIN 4 (MBD4) (Kohli and Zhang, 2013). In *Arabidopsis*, REPRESSOR OF SILENCING 1 (ROS1) and its paralogs DEMETER (DME), DEMETER-LIKE 2 (DML2) and DEMETER-LIKE 3 (DML3) are required for preventing DNA hypermethylation occurring at thousands of genomic regions (Gong et al., 2002; Law and Jacobsen, 2010). Transcript level of the *ROS1* is regulated by MET1 and some RdDM components (Huettel et al., 2006), and *ros1* mutation causes the silencing of two expression cassettes (*RD29A-LUC* and *35S-NPTII*) in a transgene (Gong et al., 2002; Zheng et al., 2008).

In DNA methylation pathway, enzymes responsible for methylating DNA are guided to specific loci by base-pairing between small RNAs and scaffold transcripts (Law and Jacobsen, 2010; He et al., 2011). Recent studies showed that the functioning of ROS1 requires a protein complex containing INCREASED DNA METHYLATION 1 (IDM1), INCREASED DNA METHYLATION 2 (IDM2), INCREASED DNA METHYLATION 3 (IDM3), and METHYL-CpG-BINDING DOMAIN 7 (MBD7) (Qian et al., 2012; Qian et al., 2014; Lang et al., 2015). Of these four proteins, the MBD7 binds to hypermethylated CG-dense region and interacts with IDM2 and IDM3 (Wang et al., 2015). Both the IDM2 and IDM3 are *α*-crystallin domain-containing protein and interact with the IDM1 (Wang et al., 2015). The *IDM1* encodes a histone acetyltransferase and such a protein is considered essential for ROS1-mediated DNA demethylation at certain loci (Wang et al., 2015). These four proteins form a functional complex and recruit ROS1 to suppress spread of DNA methylation (Wang et al., 2015). Besides, REPRESSOR OF SILENCING 3 (ROS3) was identified to bind to single-stranded RNAs and to be co-localized with ROS1 in nucleus. Moreover, both the *ros3* and *ros1* mutants exhibited concomitantly increased DNA methylation at certain loci, indicative of ROS3 acting in a same genetic pathway as did ROS1 (Zheng et al., 2008). Nevertheless, the mechanisms of how these DNA demethylases are specifically recruited to such targets remain largely unclear.

In order to gain deeper insights into the DNA demethylation pathway in Arabidopsis, in this study, we performed a genetic screening for candidate mutants with lowered LUC luminescence from an EMS (ethyl methanesulfonate)-mutagenized F_2_ population, which was derived from a parental line Col-LUC that carried a *2 × 35S-LUC* transgene and a homozygous *rdr6-11* mutation (which was able to minimize recovery of post-transcriptional gene silencing mutants) (Supplementary Figure S1). In the end, a low-LUC-luminescence mutant named *reduced LUC luminescence 6–1* (*rll6-1*) was obtained, and genome-wide DNA methylation profiling showed that mutation of *RLL6* locus led to a large number of hyper-DMRs throughout the Arabidopsis chromosomes, and there were 1,456 hyper-DMRs overlapping between *rll6-1* and *ros1-7* mutants, collectively suggesting that *RLL6* locus-encoded protein participates in both anti-silencing of a transgene and demethylation of endogenous loci through the DNA demethylation pathway.

## Results

### The *rll6-1* mutant plants showed transcriptional silencing of a *2 × 35S-LUC* transgene

**FIGURE 1:**
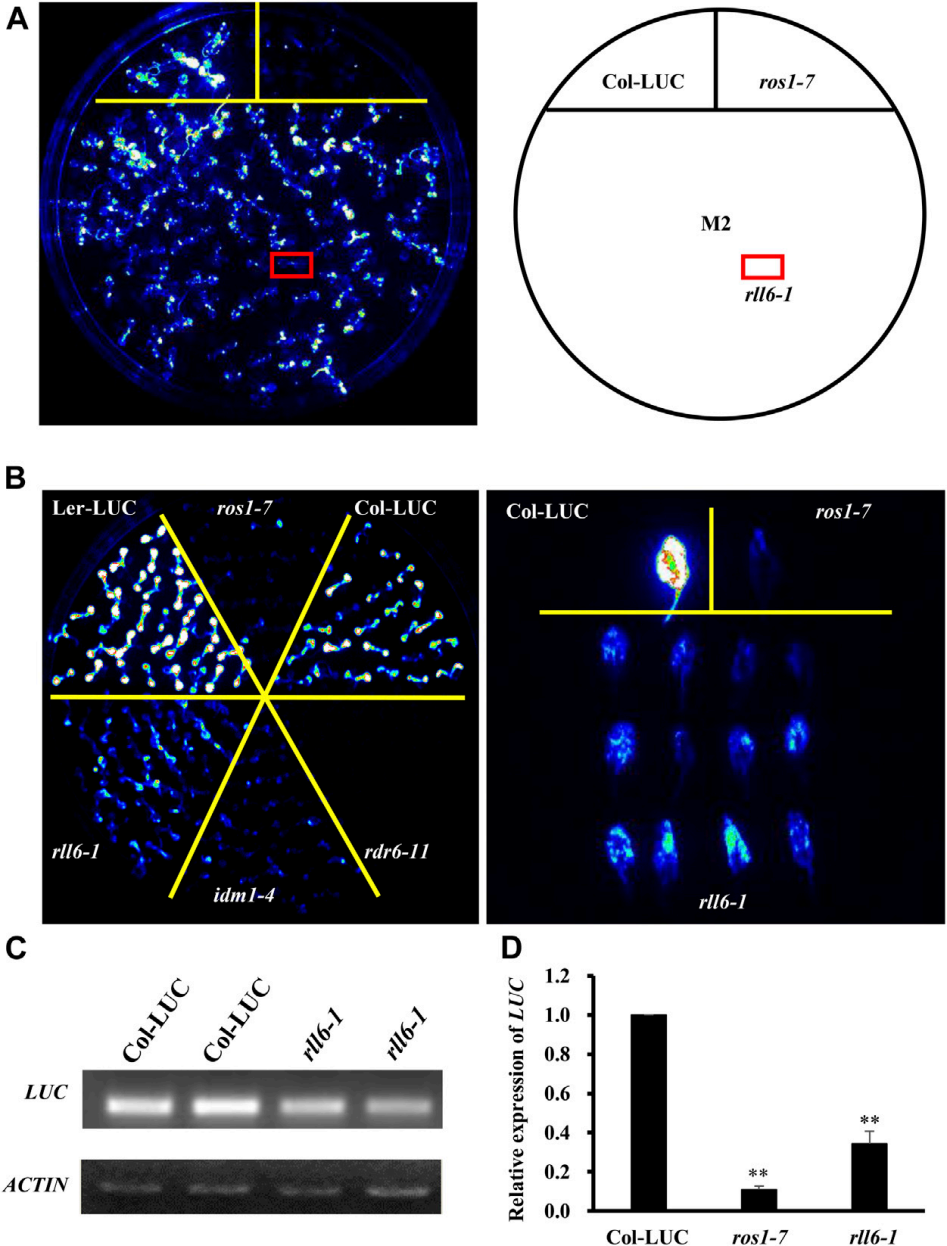
*rll6-1* mutation causes transcriptional gene silencing. **(A)** Genetic screening for low-LUC-luminescence mutants from an EMS-mutagenized M_2_ population. A candidate mutant was boxed with red and named *rll6-1*. **(B)** LUC luminescence performance of seedlings from *rll6-1* mutant compared to Col-LUC, Ler-LUC, *ros1-7* and *idm1-4* seedlings. Left panel: 10-day-old seedlings grown on 1/2 MS medium; right panel: 30-day-old detached leaves. **(C–D)** Semi-quantitative RT-PCR **(C)** and RT-qPCR **(D)** analyses of *LUC* expression in the Col-LUC and *rll6-1* mutant genotypes. The *ros1-7* mutant served as a low-LUC-luminescence control. Asterisks indicate a significant difference from the Col-LUC by Student’s *t*-test (**, *p* < 0.01). Data are the means ± SD for three biological replicates.

In order to identify more proteins participating in DNA demethylation pathway, we mutagenized a Col-LUC transgenic line by EMS and then conducted genetic screening for low-LUC-luminescence mutants; in this way, a few anti-silencing genes or DNA demethylation genes, such as *ROS1* and *IDM1*, had been recovered (Supplementary Figure S1). Our screening led to isolation of one candidate mutant, hereafter named *rll6-1*, from such a mutagenized population in the M_2_ generation (Figure 1A). When compared to the two parental lines (Col-LUC and Ler-LUC), the *rll6-1* mutant plants emitted a significantly low level of LUC luminescence, which was similar to that emitted by *ros1-7* or *idm1-4* mutant plants (Figure 1B). RT-PCR and RT-qPCR analyses showed that the expression of *LUC* gene in the *rll6-1* mutant plants was significantly reduced relative to that in Col-LUC plants, but it showed somewhat higher than in *ros1-7* mutant plants (Figures 1C,D), which was in good agreement with intensity of LUC luminescence observed in such genotypes. Thus, *RLL6* locus-encoded protein seemed to play a similar role in repressing gene silencing as did ROS1.

**FIGURE 2:**
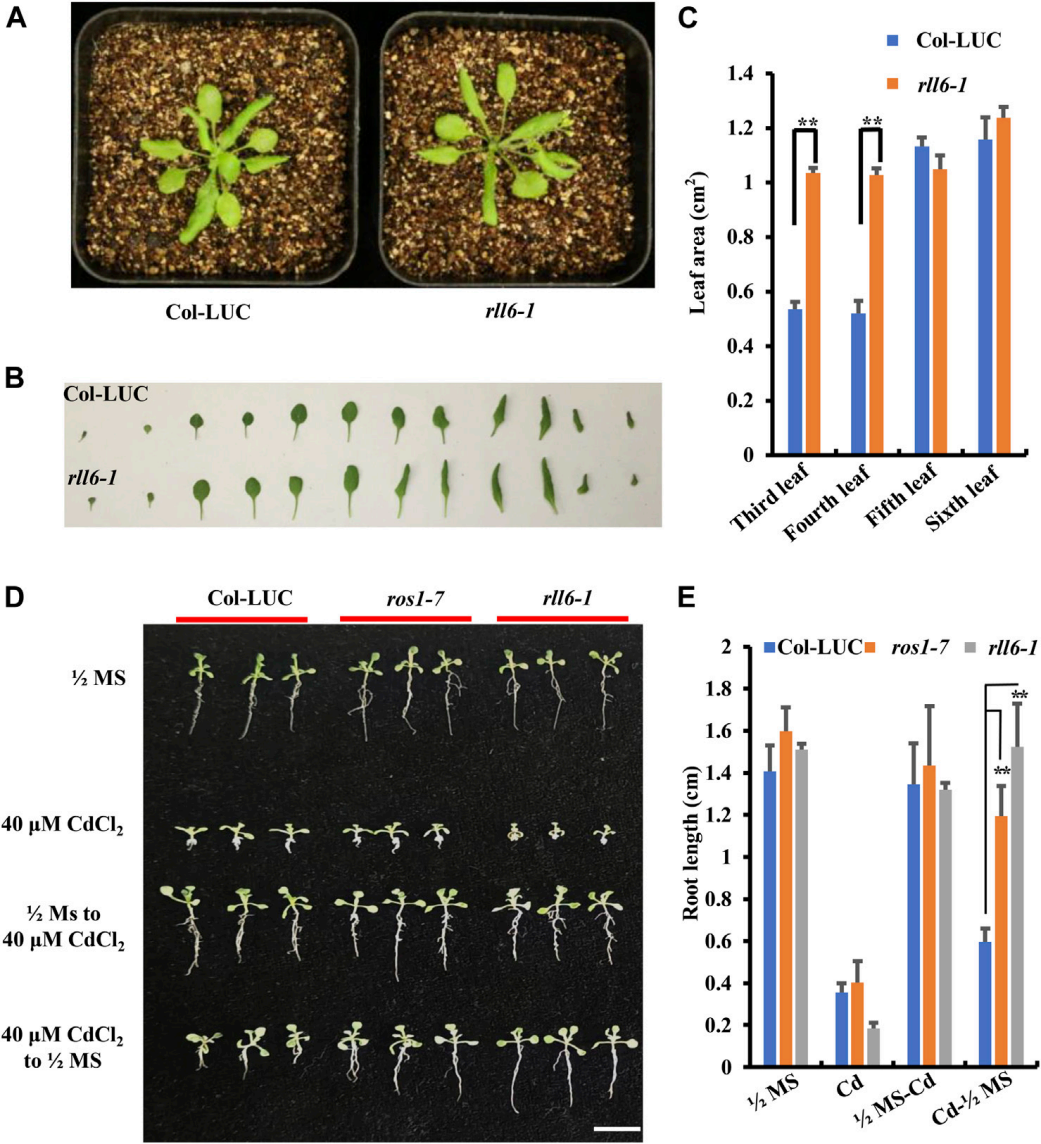
Developmental phenotypes of the *rll6-1* mutant plants under the conditions with or without a variety of abiotic stresses. **(A)** Comparisons of plant morphologies between 3-week-old *rll6-1* mutant and Col-LUC control plants. **(B)** Morphological comparisons between individual leaves from a representative *rll6-1* mutant and the counterparts from a Col-LUC plant. Both *rll6-1* mutant and Col-LUC plants were at 3 weeks of age. **(C)** Comparisons of areas of the third, fourth and fifth leaves from the *rll6-1* mutant and Col-LUC plant as shown in **(B)**. Asterisks indicate a significant difference from the Col-LUC by Student’s *t*-test (**, *p* < 0.01). **(D)** Comparisons of CdCl2 tolerance among *rll6-1* as well as *ros1-7* mutant plants and Col-LUC control plants. 1/2 MS: seeds were germinated on 1/2 MS medium, and the resulting seedlings were continuously grown on the same medium until they were photographed. 40 μM CdCl2: seeds were germinated on 1/2 MS medium supplemented with 40 μM CdCl2, and allowed the seedlings to continuously grow on the same medium until they were photographed. 1/2 MS to 40 μM CdCl2: seeds were germinated on 1/2 MS and continuously grown for 1 week, and then the seedlings were transferred to 1/2 medium supplemented with 40 μM CdCl2 to allow them to grow for another 1 week. 40 uM CdCl2 to 1/2 MS: seeds were germinated on 1/2 MS supplemented with 40 μM CdCl2 and allowed them continuously grow for 1 week, and then the seedlings were transferred to 1/2 MS medium to leave them growing for another 1 week. All seedlings were grown for a total of 2 weeks post-germination before photography. **(E)** Comparisons of lengths of roots from three genotypes (*rll6-1*, *ros1-7* and Col-LUC) that were grown on media as indicated in **(D)**. Asterisks indicate a significant difference from the Col-LUC genotype under the same treatment conditions by Student’s *t*-test (**, *p* < 0.01). Data are the means ± SD for three biological replicates.

Morphological phenotypes of the *rll6-1* mutant plants exhibited no visible differences from those of Col-LUC plants at both juvenile and bolting stages (Figure 2A and Supplementary Figure S2A); however, areas of the third and fourth leaves from *rll6-1* mutant plants were significantly larger than those from Col-LUC plants (Figures 2B,C). To know if *rll6-1* mutant plants exhibited similar MMS (methyl methanesulfonate)-sensitive phenotype as did the *ros1* mutant plants (Gong et al., 2002), the seeds from the Col-LUC and *rll6-1* mutant plants were sown on 1/2 Murashige and Skoog (MS) agar plates containing 50 mg/L MMS, and such plates were placed under normal growth conditions for 2 weeks. As shown in Supplementary Figure S2B, the *rll6-1* mutant plants did not show observable sensitivity to MMS, which was opposite to the MMS-sensitive phenotype displayed by the *ros1* mutant plants. Moreover, as was the case with MMS treatment, *rll6-1* mutant plants did also not show differences in sensitivity from Col-LUC plants when treated with NaCl or ABA (Supplementary Figure S2B).

A previous study showed that *rdd* triple mutant (for *ros1 dml2 dml3*) exhibited an elevated tolerance to CdCl_2_ stress (Fan et al., 2020). We found that, however, *rll6-1* mutant plants showed evidently enhanced sensitivity to 40 μM CdCl_2_ treatment, as shown by more retarded growth observed for the *rll6-1* mutant seedlings, when *rll6-1* mutant seeds as well as *ros1-7* and Col-LUC seeds were germinated on CdCl_2_-containing medium in parallel (Figure 2D); however, when the *rll6-1* mutant seeds were germinated on CdCl_2_-containing medium and the seedlings were then transferred to 1/2 MS, their roots grew longer than those from *ros1-7* mutant and Col-LUC plants (Figures 2D,E).

### Mapping of *rll6-1* locus

**FIGURE 3:**
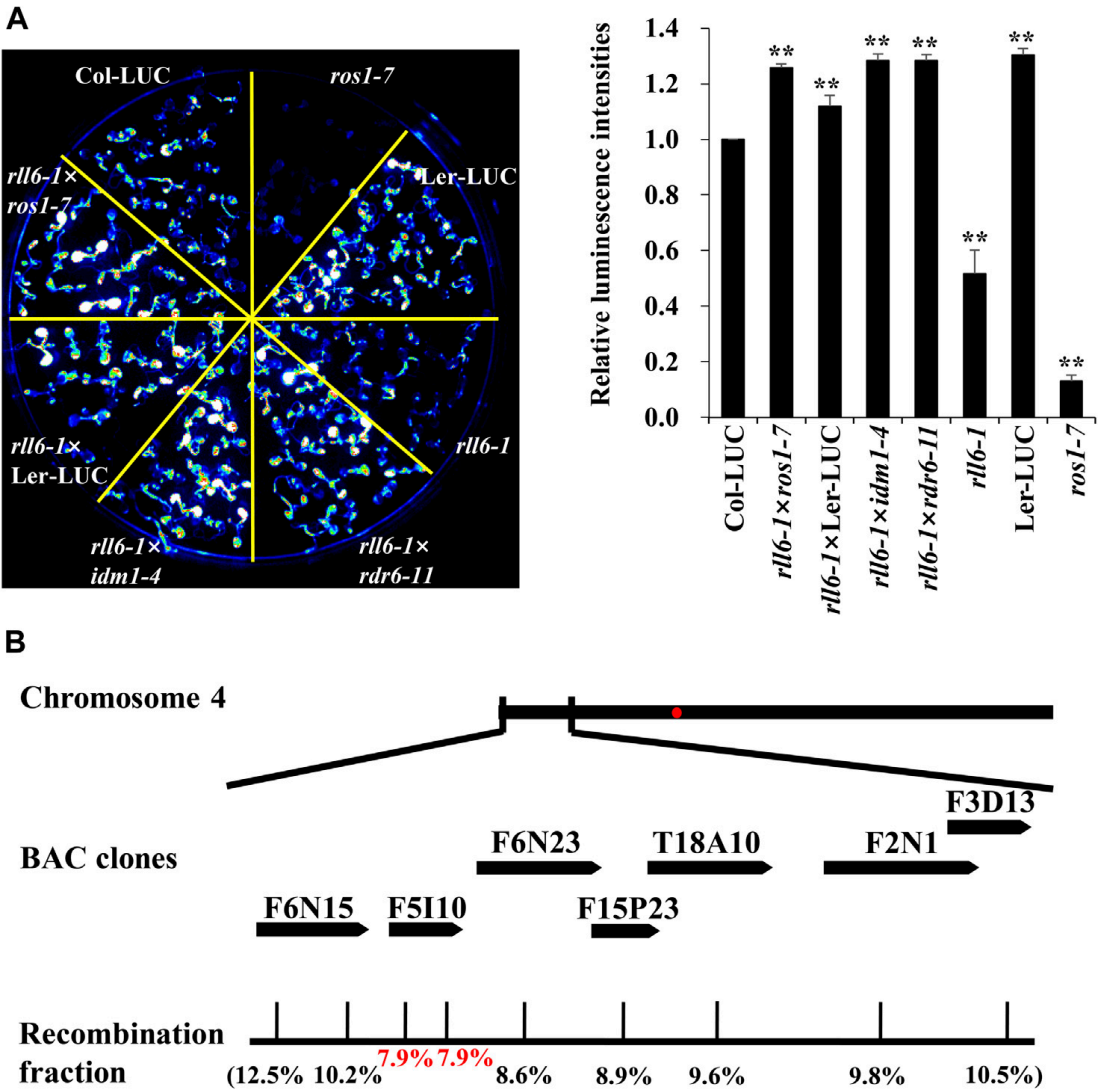
Allelic tests and map-based cloning of *rll6-1* mutation. **(A)** Left panel: Allelic tests between *rll6-1* and *ros1* or *idm1* mutant genotypes. Right panel: Relative luminescence intensities between Col-LUC and other seven genotypes. The luminescence intensities were quantified by ImageJ software. Data are the means ± SD for three biological replicates. Significant differences from Col-LUC were determined by Student’s *t*-test. **, *p* < 0.01. **(B)** Approximate location of *rll6-1* locus on Arabidopsis chromosome 4, which was identified by a map-based cloning approach. The recombination fractions in the genetic mapping were shown in parentheses.

To know if *rll6-1* locus was allelic to known mutations of genes associated with DNA demethylation pathway, *rll6-1* mutant was crossed with Ler-LUC, *ros1-7*, *idm1-4* or *rdr6-11*, respectively, to produce F_1_ seeds. The resulting F_1_ seeds were subsequently sown on 1/2 MS medium, and the seedlings were subjected to imaging of LUC luminescence following growth for 2 weeks. It was abundantly clear that the F_1_ seedlings from the crosses *rll6-1* × *ros1-7* and *rll6-1* × *idm1-4* all displayed high LUC luminescence as Col-LUC seedlings did, suggesting that *rll6-1* mutation was not allelic to *ros1* or *idm1* mutation (Figure 3A). The low-LUC-luminescence phenotype of the *rll6-1* mutant plants did also not result from mutation of the *LUC* gene because the seedlings from the cross *rll6-1* × *rdr6-11* (*rdr6-11* mutant did not carry *2 × 35S-LUC* transgene) exhibited obviously increased LUC luminescence in comparison with those from *rll6-1* mutant seedlings (Figure 3A). Altogether, these results indicated that *RLL6* locus appears to encode a protein that participates in anti-silencing of a transgene.

To map *rll6-1* locus, we crossed the *rll6-1* mutant plants with Ler-LUC plants, and the resulting F_2_ population was used for such a purpose. It was evident that the F_2_ individuals showed a phenotype segregation of 3:1 (high-LUC-luminescence: low-LUC-luminescence = 408:120; χ^2^ = 1.34), implicating that the *rll6-1* mutant carried a recessive mutation occurring in a single nuclear gene. Primary mapping was conducted by selecting 120 stably-low-LUC-luminescence plants for genetic linkage analysis (Supplementary Figure S3A). The results indicated that the rll6-1 locus was mapped to the short arm of chromosome 4, which was in the vicinity of BAC clone F6N15 (Supplementary Figure S3B). For fine mapping the *rll6-1* locus, 386 low-LUC-luminescence plants out of 1659 F_2_ progenies were used. Eventually, the *rll6-1* locus was narrowed down to an ∼80-kb region within the BAC clone F5I10 (Figure 3B). Gene annotation from TAIR website revealed that there were 10 candidate genes residing in such a region (Table 1), which encoded proteins of different molecular functions, including transcriptional regulation, carbohydrate binding, methylated CpG binding, RNA binding, and so on. Further studies are needed to determine which one is the true gene responsible for maintenance of LUC luminescence.

**TABLE 1:** Ten candidate genes residing within the mapping region.

Gene Locus	Functional Annotation
AT4G00232	DNA-binding storekeeper protein-related transcriptional regulator
AT4G00280	ER protein carbohydrate-binding protein
AT4G00295	fringe-like protein
AT4G00305	RING/U-box superfamily protein
AT4G00342	hypothetical protein
AT4G00390	DNA-binding storekeeper protein-related transcriptional regulator
AT4G00416	MBD3, Protein containing methyl-CpG-binding domain
AT4G00420	Double-stranded RNA-binding domain-containing protein
AT4G00480	MYC1, MYC-related protein with a basic helix-loop-helix motif at the C-terminus
AT4G00540	MYB3R2, Encodes a putative c-myb-like transcription factor

### *rll6-1* mutation led to an increase of DNA methylation at a few ROS1-targeted loci

**FIGURE 4:**
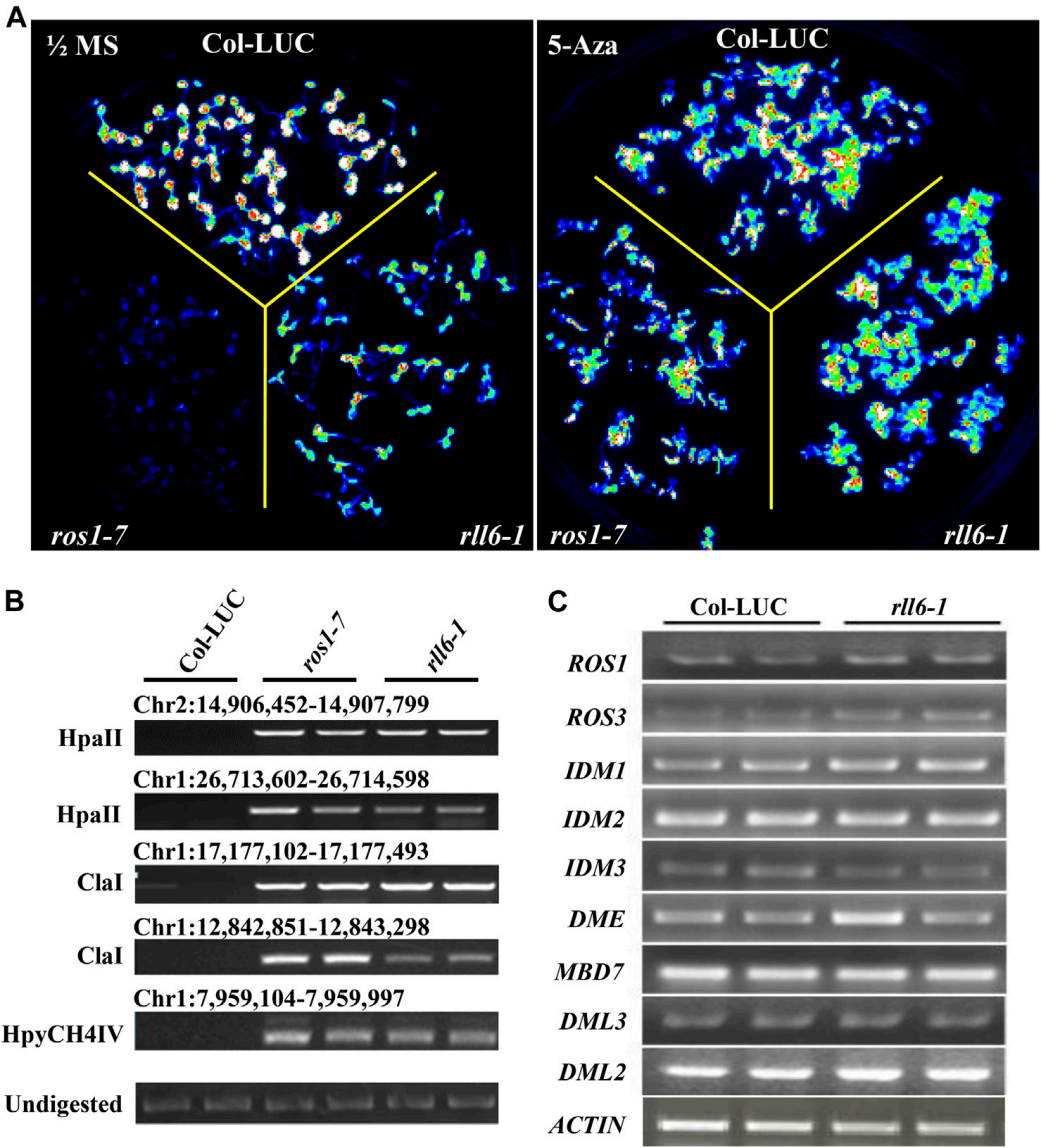
Effects of 5-Aza-2′-deoxycytodine on *LUC* expression in *rll6-1* mutant plants. **(A)** Performance of LUC luminescence of 14-day-old seedlings from *rll6-1*, *ros1-7* and Col-LUC genotypes under the conditions with or without 7 μg/ml 5-Aza-2′-deoxycytodine treatments. Left panel: image of LUC luminescence from seedlings of the three genotypes as indicated grown on 1/2 MS medium for 1 week. Right panel: image of LUC luminescence from seedlings of three genotypes as indicated grown on 1/2 MS medium, which was supplemented with 7 μg/ml 5-Aza-2′-deoxycytodine, for 1 week. **(B)** Examination of DNA methylation status at several endogenous loci in *ros1-7* and *rll6-1* mutants by Chop-PCR method. HpaII, ClaI and HpyCH4IV were methylation-sensitive restriction enzymes used to digest genomic DNA. Undigested DNA served as a control. **(C)** Detection of expression levels of several essential genes, which were involved in ROS1-mediated DNA demethylation pathway, in *rll6-1* mutant by semi-quantitative RT-PCR method.

To know whether the silencing of *LUC* transgene in *rll6-1* mutant plants resulted from enhanced DNA methylation occurring on the promoter of such a transgene, *rll6-1* mutant seedlings were treated with DNA methyltransferase inhibitor 5-Aza-2′-deoxycytodine which was known to cause global DNA hypomethylation when they were used to treat plants. It was apparent that the LUC luminescence emitted from the *rll6-1* mutant seedlings was obviously enhanced after the treatment (which was similar to that emitted from Col-LUC seedlings), as it was in *ros1-7* (Figure 4A). Therefore, this result suggested that mutation of *RLL6* locus very likely led to an elevation of DNA methylation level on the *2 × 35S* promoter situated in front of *LUC* gene.

In order to find if mutation of *RLL6* locus results in increased DNA methylation levels at endogenous genomic loci as well, we examined the DNA methylation status of a few particular loci (which showed noticeable hypermethylation in *ros1-7* mutant) in *rll6-1* mutant by using the Chop-PCR method (Figure 4B). It was interesting that the *rll6-1* mutant, as did the *ros1-7*, exhibited elevated level of DNA methylation at the aforementioned loci, suggesting that *RLL6* locus-encoded protein presumably works in close collaboration with ROS1 to counteract DNA methylation in such loci. To ascertain if the increases of DNA methylation levels at those loci were the result of downregulation of *ROS1* expression, we examined the expression of genes involved in ROS1-mediated DNA demethylation pathway in the *rll6-1* mutant by RT-PCR assays. Surprisingly, there were no significant expression changes for the nine genes detected, such as *ROS1*, *ROS3*, *IDM1*, *DME* and *MBD7*, etc., in the *rll6-1* mutant plants (Figure 4C), clearly indicating that the elevated DNA methylation at certain loci caused by mutation of *RLL6* locus may be not a result of deficiency or downregulation of the expression of DNA demethylation pathway genes.

### Mutation of *RLL6* locus brought about genome-wide DNA hypermethylation as did *ros1* mutation

**FIGURE 5:**
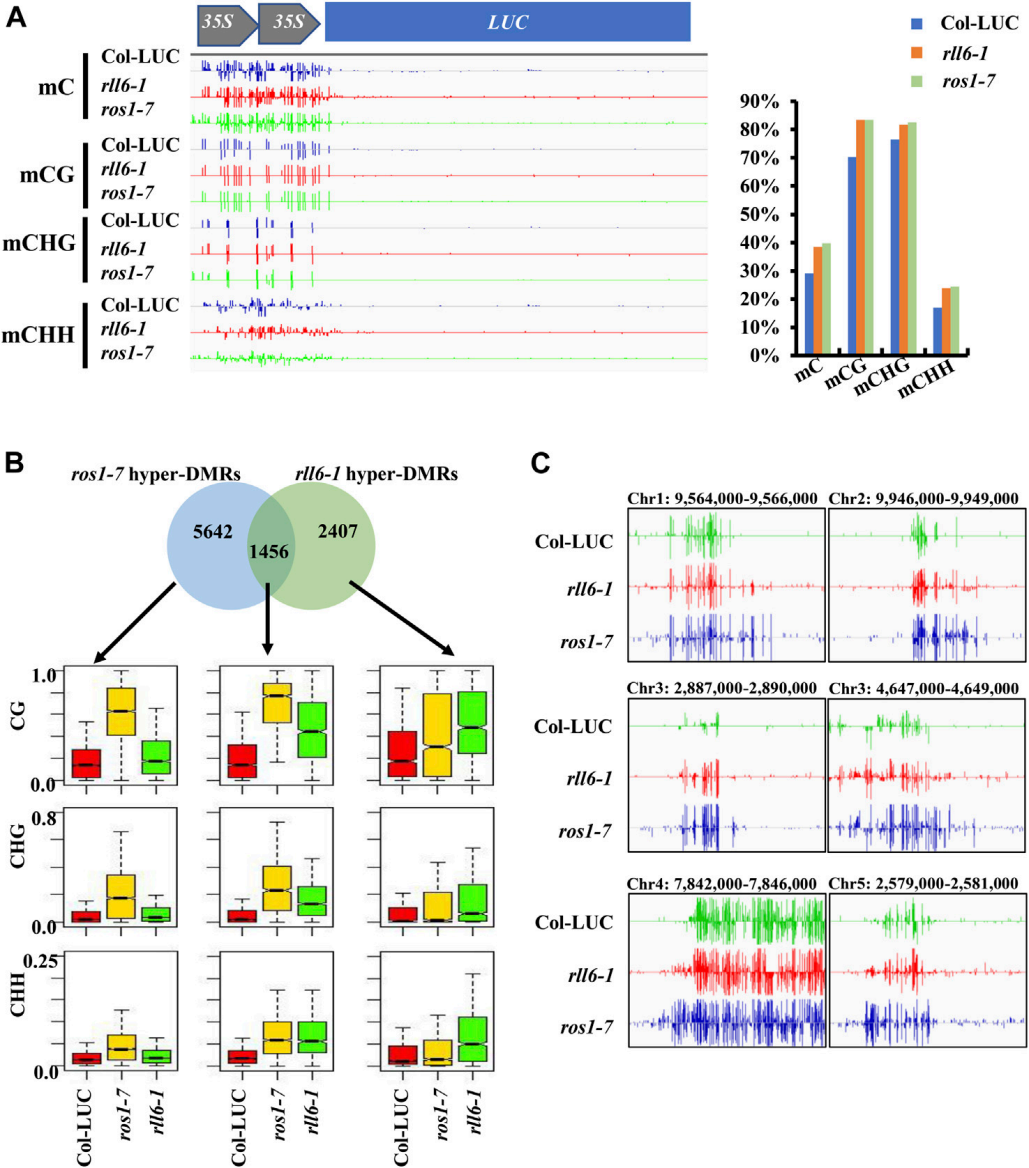
Analysis of hyper-DMRs identified in *rll6-1* and *ros1-7* mutants. **(A)** DNA methylation levels of *2 × 35S* promoter in *rll6-1* as well as *ros1-7* mutant and Col-LUC control genotypes. Left panel: a screenshot of DNA methylation status in *2 × 35S* promoter regions from the three genotypes as indicated. Right panel: quantification of methylation levels of the *2 × 35S* promoter regions shown in **(A)**. **(B)** Overlap of hyper-DMRs between the *rll6-1* and *ros1-7* mutants. Boxplots showed methylation levels (for CG, CHG and CHH sequence contexts) of each class of hyper-DMRs (1,456 for overlapped hyper-DMRs; 5,642 and 2,407 for hyper-DMRs unique to *ros1-7* and *rll6-1* mutants, respectively). **(C)** Screenshots of DNA methylation status of several endogenous genomic loci from the *rll6-1* as well as *ros1-7* mutant and Col-LUC control genotypes.

Given the fact that a few genomic loci, which showed increased DNA methylation in *ros1-7* mutant, exhibited prominent DNA hypermethylation in *rll6-1* mutant, we wondered whether there were more DNA hypermethylated loci shared by both mutants on a genome-wide scale. Therefore, both mutants were subjected to the whole-genome bisulfite sequencing. Analyses of DNA methylation profiling data revealed noticeable increases in DNA methylation in three sequence contexts (CG, CHG, and CHH) on the *2 × 35S* promoters in the *rll6-1* mutant; moreover, the levels of DNA methylation in each of the three sequence contexts were virtually the same between *rll6-1* and *ros1-7* mutants (Figure 5A). Further analysis indicated that genome-wide DNA hypermethylation occurred at the three sequence contexts (Supplementary Figure S4A), and the percentage proportional distributions of hyper-DMRs on each of constituents of genome [gene, TE, and intergenic region (IG)] were quite similar between the rll6-1 and ros1-7 mutants, with approximately 38% of hyper-DMRs concentrated on TEs in both mutants (Supplementary Figure S4B); furthermore, it was remarkable that the largest proportion of the hyper-DMRs overlapping with TEs appeared to cluster on 0–0.5-kb TEs in both the mutants (Supplementary Figure S4C). The analyses also demonstrated that there was a total of 3,863 hyper-DMRs and 700 hypomethylated differentially methylated regions (hypo-DMRs) were identified in the rll6-1 mutant relative to Col-LUC genotype, while 7,098 hyper-DMRs and 410 hypo-DMRs were identified in the ros1-7 mutant versus the same Col-LUC plant (Supplementary Table S1). We then compared the hyper-DMRs between *rll6-1* and *ros1-7* mutants and obtained 1,456 common hyper-DMRs, which accounted for about 37.7% and 20.5% of total hyper-DMRs in the *rll6-1* and *ros1-7* mutants, respectively (Figure 5B). Boxplot analysis indicated that for such 1,456 common hyper-DMRs, their methylation levels in the three sequence contexts were all obviously increased in both mutant genotypes by comparison with Col-LUC genotype (Figure 5B); for example, a few loci (chr1: 9,564,000–9,566,000, chr2: 9,946,000–9,949,000, chr3: 2,887,000–2,890,000, chr3: 4,647,000–4,649,000, etc.) showed clear hypermethylation in the *rll6-1* and *ros1-7* mutants, when compared to the Col-LUC control (Figure 5C). Moreover, as for the hyper-DMRs unique to *rll6-1* mutant, the elevation of DNA methylation levels in the three sequence contexts in the *rll6-1* mutant genotype versus Col-LUC genotype was coincident with the increase of those in *ros1-7*; however, as regards the 5,642 hyper-DMRs exclusive to the *ros1-7*, although the rise of those in the *ros1-7* mutant genotype versus the Col-LUC genotype was significant, the methylation levels in the three sequence contexts in the *rll6-1* mutant genotype was just marginally increased compared to the Col-LUC genotype (Figure 5B). Taken together, these results further supported the above-mentioned notion that the *RLL6* locus-encoded protein presumably acts in close collaboration with ROS1 to antagonize DNA methylation in thousands of loci at a genome-wide level.

## Discussion

In this study, we identified one new low-LUC-luminescence mutant, and mapped the mutation to a region in bacterial artificial chromosome (BAC) clones F5I10 on Arabidopsis chromosome 4 (Figures 1, 3). Our mapping of *rll6* locus led to the identification of a region including 10 candidate genes (Table 1). Among the 10 genes, the *MBD3* (AT4G00416), *MYB3R2* (AT4G00540) and double-stranded RNA-binding domain-containing protein (AT4G00420) appeared to be quite interesting. There are 13 Methyl-CpG-binding domain-containing proteins present in Arabidopsis genome, three of which, i.e. MBD5, MBD6 and MBD7, were found to bind specifically to methylated CG sites *in vitro* (Zemach and Grafi, 2007). Recent studies showed that MBD7 was physically associated with the histone acetyltransferase IDM1, and it participated in active DNA demethylation in Arabidopsis (Lang et al., 2015; Wang et al., 2015). Thus, MBD3 seems to be a potential candidate involved in inhibiting DNA methylation and preventing transcriptional gene silencing. MYB proteins are known to generally function as transcription factors engaging in the defense responses of plants (Zheng et al., 2012). In Arabidopsis, MYB74 belongs to the R2R3-MYB protein family, and such a protein was transcriptionally regulated by RdDM pathway (Xu et al., 2015). As a putative c-myb-like transcription factor, whether the MYB3R2 (AT4G00540) is involved in the RdDM pathway or DNA demethylation pathway remains unclear. Moreover, the double-stranded RNA-binding domain-containing protein (AT4G00420) appeared to be also a candidate because, previous research showed that an RNA-binding protein ROS3 was required for DNA demethylation pathway in Arabidopsis (Zheng et al., 2008); hence, such a protein is worth being further examined to find whether it participates in the DNA demethylation pathway.

Previous research showed that there existed the same and different mechanisms underlying the silencing of *2 × 35S* promoter when compared to *RD29A* promoter (Gong et al., 2002). Using the same genetic screening system, a *ros1-7* mutant allele was identified, in which the increased DNA methylation level on the *2 × 35S* promoter resulted in transcriptional silencing of *LUC* gene (Hou et al., 2022). However, *SAC3B* dysfunction resulted in *LUC* gene silencing without obviously altering promoter DNA methylation level of the transgene *2 × 35S-LUC* (Yang et al., 2017). Our study revealed that *RLL6* locus-encoded protein was also required to protect the *2 × 35S* promoter from being silenced just like ROS1 did, so the two anti-silencing factors seemed to play similar roles in anti-silencing of such a promoter as DNA methylation patterns at the *2 × 35S-LUC* promoter were increased both in the *ros1* and in *rll6-1* mutants (Figure 5A). Moreover, the silencing state of such a promoter in *rll6-1* mutant seedlings could be released by 5-Aza-2′-deoxycytodine, similar to what was observed in *ros1-7* seedlings (Figure 4A). Therefore, these results indicate that both *RLL6* locus-encoded protein and ROS1 participate in inhibiting transcriptional gene silencing through a similar mechanism.

Epigenetic mutations are usually accompanied by developmental defects in Arabidopsis. DEMETER (DME) demethylated small transposons and edges of long transposons (Gehring et al., 2006; Schoft et al., 2011; Ibarra et al., 2012; Park et al., 2017; Frost et al., 2018), and mutation of *DME* resulted in seed abortion and abnormal germination of pollen tubes (Choi et al., 2002; Schoft et al., 2011). Mutation of *increase IN BONSAI METHYLATION 1* (*IBM1*) induced a variety of developmental phenotypes, including smaller leaves, abnormal flower development and reduced fertility, which depended on methylation status of histone H3 at lysine 9 (Saze et al., 2008). Arabidopsis SAC3B dysfunction caused elevation in the repressive histone mark H3K9me2, accompanied by shorter roots, smaller leaves and shorter inflorescence (Yang et al., 2017). In this study, we observed that *rll6-1* mutant showed enlarged third and fourth leaves, although the effect was weaker than that in other mutants mentioned above, implicating that *RLL6* locus-encoded protein may be also involved in the regulation of plant development (Figures 2A,B).

One of the most important roles of DNA methylation was to silence TEs, which exist extensively in plant and animal genomes and tend to spread to adjacent genes (Bennetzen and Wang, 2014). Previous research showed that transcription of *EPF2* gene, which was near a methylated TE in Arabidopsis, relied on the demethylation of ROS1, which played a critical role in preventing spread of DNA methylation from methylated TEs to adjacent sequences (Yamamuro et al., 2014). Our findings revealed that *RLL6* locus-encoded protein also limited the spread of DNA methylation from high methylated regions to neighboring regions (Figure 5C).

In plants, active DNA demethylation is initiated by the ROS1/DME family of 5-methylcytosine DNA glycosylases (He et al., 2009). In this study, many hypermethylated loci were identified in *rll6-1* mutant, and more than one third were overlapped with those of *ros1-7* mutant (Figure 5B). Moreover, both mutants showed similar increased DNA methylation patterns in many representative loci, suggesting that both proteins synergistically regulated DNA hypermethylation in some loci (Figure 5B). To date, how the ROS1 was targeted to speciﬁc genomic loci remains largely unclear. Our data indicated that *RLL6* locus-encoded protein had a very close relationship with ROS1, whereas mutation of the *RLL6* locus did not affect expression of *ROS1* as well as other ROS1/DME family genes (Figure 4C). Recent research showed that the IDM1-IDM2-IDM3-MBD7 complex played an important role in facilitating active DNA demethylation through ROS1 (Lang et al., 2015; Wang et al., 2015); however, mutation of *RLL6* locus did not affect the expression of these genes (Figure 4C), demonstrating that *RLL6* locus-encoded protein inhibited DNA hypermethylation not by affecting the expression of those genes participating in ROS1-mediated DNA demethylation. Moreover, the 2,407 unique hyper-DMRs existing in the *rll6-1* mutant indicated that the *RLL6* locus-encoded protein could inhibit DNA hypermethylation independently of ROS1 (Figure 5B). So, it is necessary to pinpoint the mutation site on *RLL6* locus to further understand the roles of the *RLL6* locus-encoded protein in ROS1-dependent DNA demethylation pathway and ROS1-independent DNA demethylation process.

## Materials and methods

### Plant materials and growth conditions

The wild-type plants in this study were from the Col-LUC transgenic line bearing *2 × 35S-LUC* in the *rdr6-11* mutant background. The Ler-LUC line was the Landsberg-0 (Ler-0) plant harboring the same *2 × 35S-LUC* transgene and *rdr6-11* mutation as those present in Col-LUC line, which was generated by backcrossing Col-LUC with Ler-0 six times. All the seeds we used were surface-sterilized with 0.8% sodium hypochlorite (NaClO) before sown on Murashige and Skoog (MS) medium containing 0.8% (w/v) agar and 2% (w/v) sucrose. For NaCl, ABA, MMS, CdCl_2_ or 5-Aza-2′-deoxycytodine treatments, seeds were sown on 1/2 MS medium supplemented with 0.8% (w/v) agar and 2% (w/v) sucrose, with 50 mg/L MMS, 0.1 M NaCl, 0.2 μM ABA, or 7 μg/ml 5-Aza. Each treatment was repeated three times (three replicates), and 150 seeds were sown for each replicate. After vernalization at 4°C for 48 h, the plants were transferred to a long-day photoperiod (16-h light/8-h dark) at 22°C in a growth room. EMS mutagenesis and screening of mutants with reduced LUC luminescence were conducted as described previously (Yang et al., 2017).

### Imaging of LUC luminescence

For LUC luminescence imaging, seedlings were sown on 1/2 MS medium, which contained 0.8% (w/v) agar and 2% (w/v) sucrose. Prior to the imaging, two-week-old seedlings were placed in the dark for 5 min after being sprayed with 1 mM luciferin. Then the plants were put in a Princeton Dark Box equipped with a Roper VersArray1300B camera controlled by the WinView32 software, and was imaged with a 30-s exposure time.

### Map-based cloning

To map the *rll6-1* mutation, *rll6-1* mutant plants (female) were crossed with Ler-LUC plants (male), and the resulting F_1_ seeds were selfed to produce an F_2_ population. The low-LUC-luminescence F_2_ individuals were selected to form a mapping population. Primary mapping with 120 F_2_ individuals delimited the *rll6-1* locus to the top of the chromosome four in the vicinity of the BAC clone F6N15. Fine mapping further narrowed down the *rll6-1* locus to an ∼80-kb region on the BAC clone F5I10 by using 386 F_2_ individuals.

### Analysis of gene expression

Gene expression analysis was carried out according to the methods described previously (Lei et al., 2014). Briefly, RNA was extracted from 14-days-old seedlings, and about 1 μg of total RNA was used to synthesize the first-strand cDNA using One-step gDNA removal and cDNA Synthesis SuperMix kits (Transgen, Beijing, China). Subsequently, the cDNA was used for RT-PCR or qRT-PCR analysis for *LUC* expression; a housekeeping gene *ACTIN2* served as internal controls for all reactions.

### Analysis of DNA methylation levels

For Chop-PCR assays, approximately 600 ng of genomic DNA was digested by methylation-sensitive enzymes for 16 h, and then they were subjected to PCR with different primer sets. All the primers used were listed in the Supplementary Table S2. For whole-genome bisulfite sequencing, DNA samples were extracted from two-week-old seedlings by the CTAB method, then used for bisulfite treatment and DNA sequencing [Novogene (Beijing, China)] as previously described (Duan et al., 2015). Differentially methylated regions (DMRs) were identified as described previously (Duan et al., 2015).

## Data Availability

The datasets presented in this study can be found in online repositories. The names of the repository/repositories and accession number(s) can be found in the article/Supplementary Material.

## Abbreviations

AID/APOBEC, activation induced deaminase/apolipoprotein B RNA-editing catalytic component-1; BAC, bacterial artificial chromosome; CMT2, Chromomethylase two; CMT3, Chromomethylase three; DRM2, domains rearranged methylase two; CpG-binding domain four; DMRs, differentially methylated regions; DME, demeter; DML2, demeter-like two; DML3, demeter-like three; EMS, ethyl methanesulfonate; rll6-1, *reduced LUC luminescence six to one*; \5-meC, 5-methylcytisine; MMS, methyl methanesulfonate; MS, Murashige and Skoog; MET1, Methyltransferase one; MBD4, methyl-ROS1, repressor of silencing one; IDM1, increased DNA methylation one; RdDM, RNA-directed DNA methylation; IDM2, increased DNA methylation two; IDM3, increased DNA methylation three; MBD7, methyl-CpG-binding domain seven; ROS3, repressor of silencing one; rdd, ros1 dml2 dml3 triple mutant; IG, intergenic region; IBM1, Increase in bonsai methylation one; TDG, thymine DNA glycosylase; TET, ten-eleven-translocation; TFEB, transcription factor EB
