# Supplementary material for: Fine mapping and characterization of RLL6 locus required for anti-silencing of a transgene and DNA demethylation in Arabidopsis thaliana
Source: Front Genet. 2022 Sep 26;13:1008700. doi: 10.3389/fgene.2022.1008700 (PMC9549997; doi:10.3389/fgene.2022.1008700)
Supplement: Supplementary file 1 [file DataSheet2.PDF]

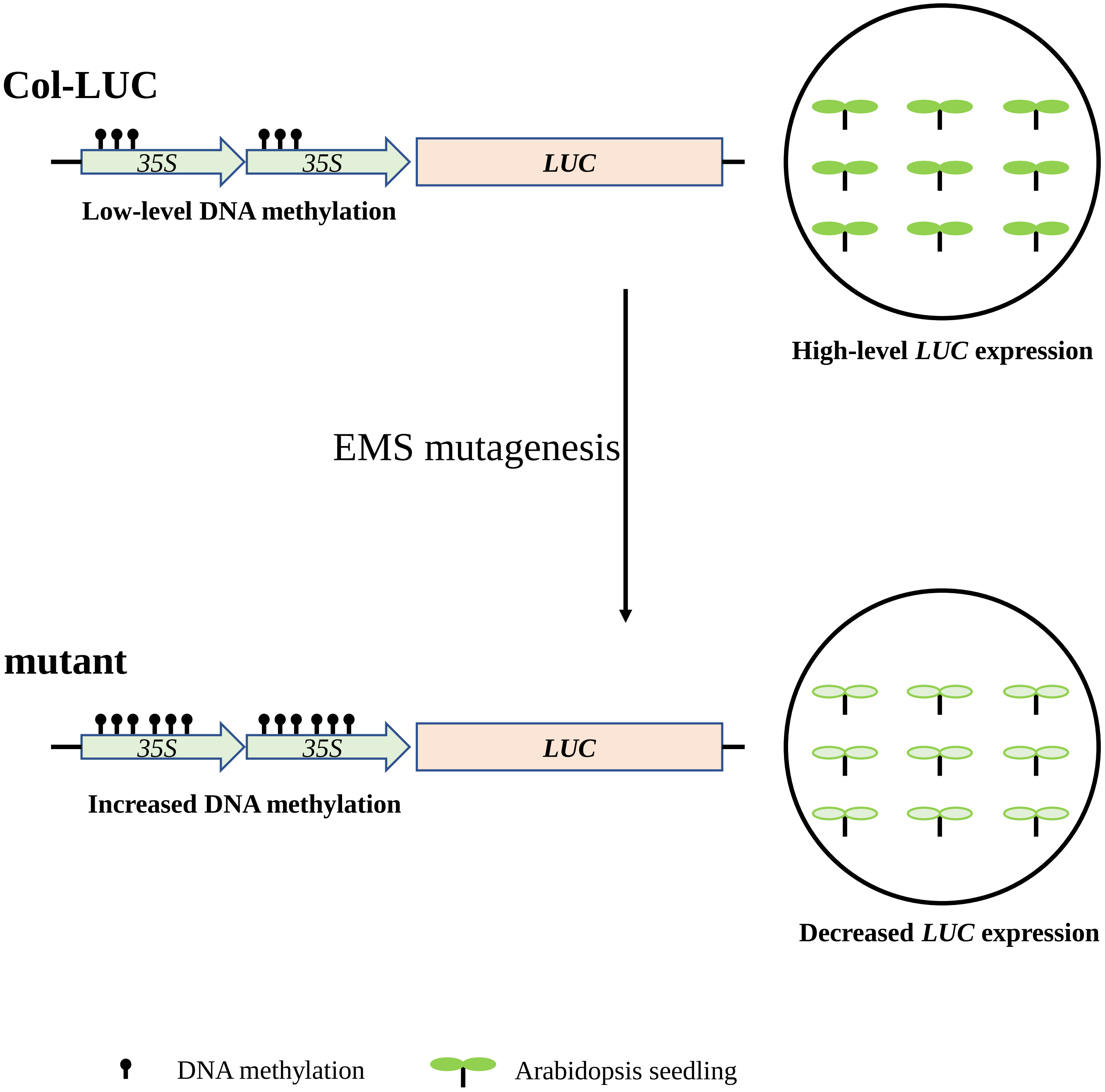

Supplementary Figure S1 | A schematic diagram of the genetic screening system for identifying low-LUC-luminescence mutants. The Col-LUC parental line is in the *rdr6-11* mutant background.

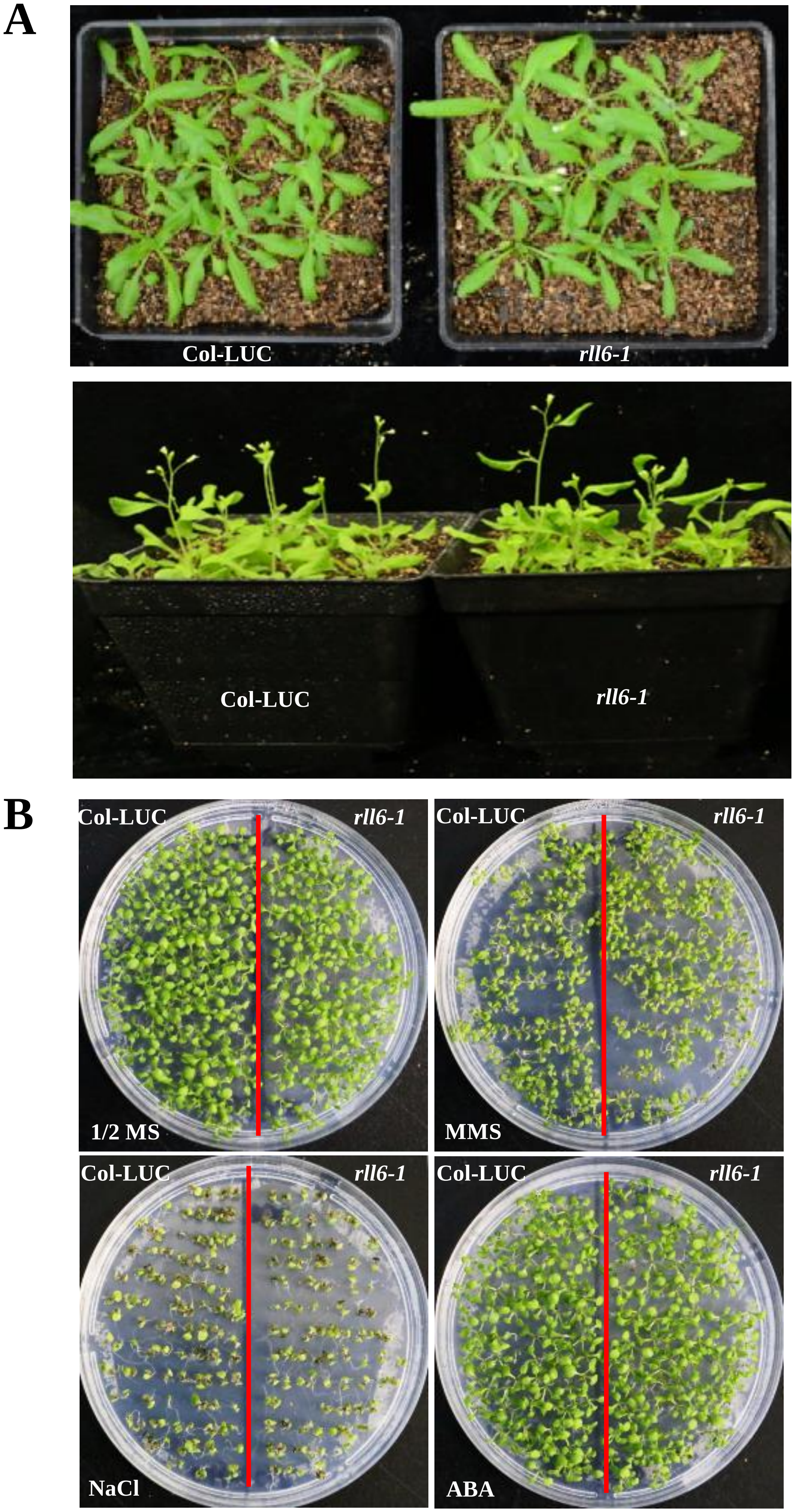

Supplementary Figure S2 | Developmental phenotypes of *rll6-1* mutant plants under the conditions with or without different abiotic stresses. (A) Developmental phenotypes of *rll6-1* mutant plants at the juvenile (3-week-old) and bolting (5-week-old) stages. **(B)** Responses of *rll6-1* mutant plants to treatments by 50 mg/L MMS, 0.1 M NaCl, or 0.2 μM ABA. Seeds of the *rll6-1* and Col-LUC mutants were germinated on medium containing one of chemicals as indicated, and the resultant seedlings were left continuously growing on the same media for 10 days.

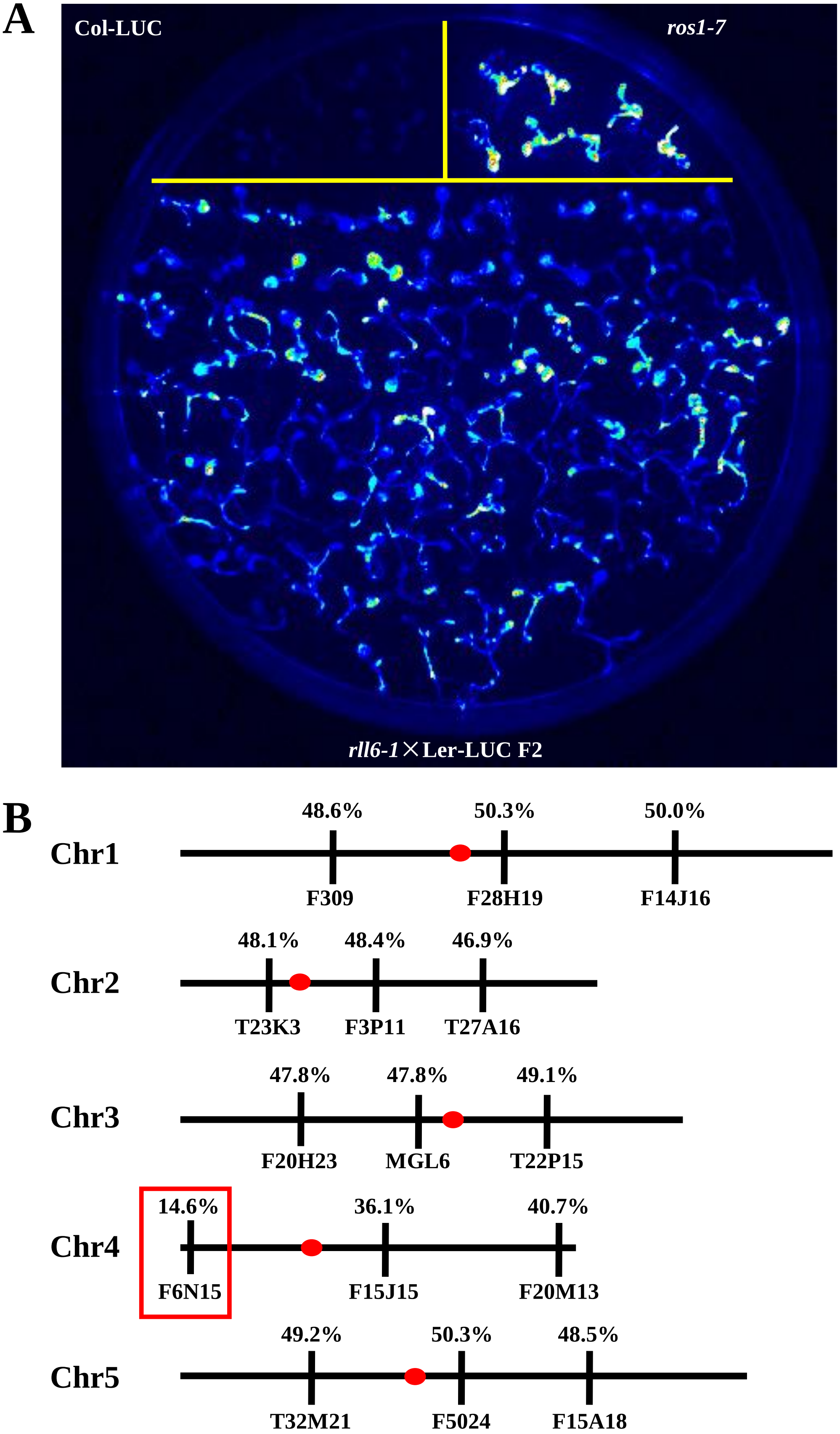

Supplementary Figure S3 | Primary mapping of *rll6-1* mutation. (A) Image of LUC luminescence of the F_2 individuals used for primary mapping, which were derived from a mapping population generated from a cross between *rll6-1* mutant and Ler-LUC plants. **(B)** Recombination frequencies occurring between adjacent markers within the indicated BAC clones on five chromosomes when the low-LUC-luminescence F_2 individuals were chosen and used for primary mapping. The region boxed in red was where the *rll6-1* mutation resides.

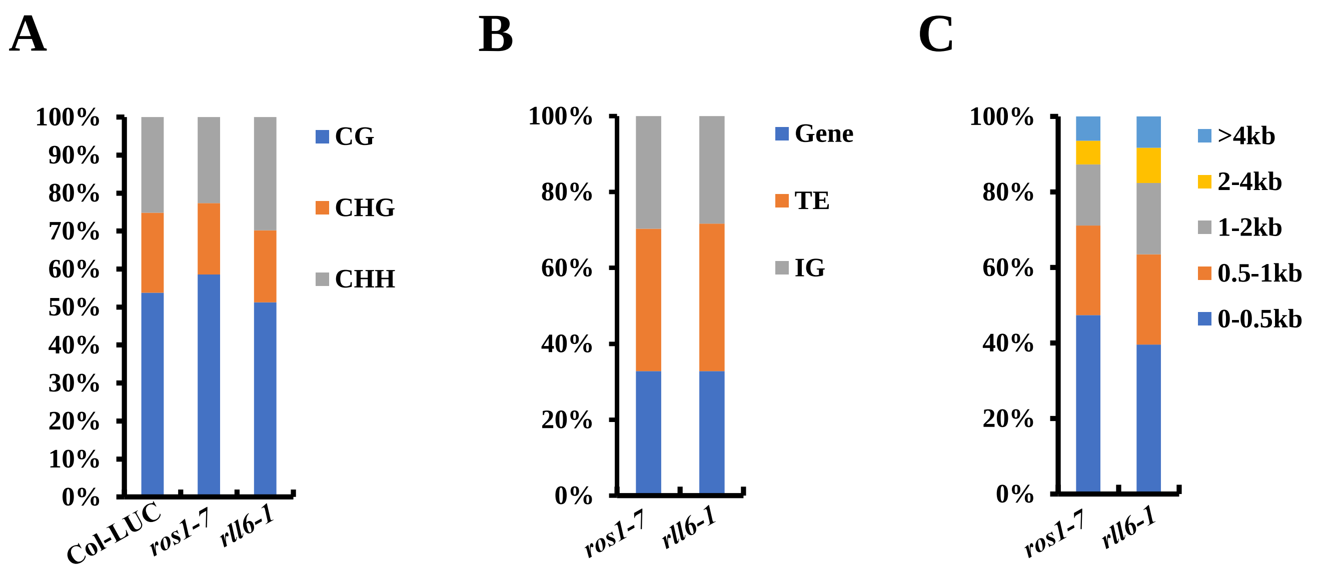

Supplementary Figure S4 | Proportional Distributions of hyper-DMRs in *rll6-1* and *ros1-7* mutants. (A) Percentage occurrence of DNA hypermethylation at CG, CHG and CHH sequence contexts in the indicated three genotypes, Col-LUC, *ros1-7* and *rll6-1*. **(B)** Percentage proportional distributions of hyper-DMRs in gene, transposable element (TE) and intergenic region (IG) in *rll6-1* and *ros1-7* mutants. **(C)** Percentage proportional distributions of hyper-DMRs located on TEs in different size groups in *rll6-1* and *ros1-7* mutants.
