# Supplementary material for: Fine mapping and characterization of RLL6 locus required for anti-silencing of a transgene and DNA demethylation in Arabidopsis thaliana
Source: Front Genet. 2022 Sep 26;13:1008700. doi: 10.3389/fgene.2022.1008700 (PMC9549997; doi:10.3389/fgene.2022.1008700)
Supplement: Supplementary file 2 [file DataSheet1.PDF]

Table S1. The hyper-DMRs and hypo-DMRs in *rll6-1* and *ros1-7* mutants

Genotype	Hyper-DMRs	Hypo-DMRs
rll6-1	3863	700
ros1-7	7098	410

Table S2. Primers used in this study

Primer names	primer sequence
CL105_F309-F	GTACAGCGCGGGCCCTTCGTT
CL105_F309-R	TGTCGGGCAGATAAACTATAA
CL116_F28H19-F	TGCGGGAGTGTGATAGAATA
CL116_F28H19-R	TCCTCGAAAGATTCATTGAT
CL121_F14J14-F	CCGATAATAGGAGTTTGTCCCA
CL121_F14J14-R	GGTACATCCGACATGCATGA
CL200_T23K3-F	CGTGTTTACCGGGTCGGA
CL200_T23K3-R	AAAACCCTTGAAGAATACG
CL208_F3P11-F	ATGTATTTGTTGCAAAATAA
CL208_F3P11-R	TGCACAGAAGAAAAAACTA
CL212_T27A16-F	ATGAACGGAGTAGCTATC
CL212_T27A16-R	CGCGTAGAACATAATCTGTA
CL301_F20H23-F	CAATGGGAAGAAGGTGTGAG
CL301_F20H23-R	CGCATTTCCATAAGTTTGTT
CL305_MGL6-F	ACCTGTTCAGTCTATGTTAC
CL305_MGL6-R	GGGAATTATTAACATTATCA
CL312_T22P15-F	GGCGGAAGCGGTGTGGCTTG
CL312_T22P15-R	CGGCTCTTAATCAAGTGTACTCGC
CL400-F6N15-F	CATTAAAATCACCGCCAAAAA
CL400-F6N15-R	TTTTGTTACATCGAACCACACA
CL410_F15J15-F	CTAAGCGGGTCGGGTCGATTCG
CL410_F15J15-R	GAACGCTCCTTTTAATCAGACCG
CL418_F20M13-F	GAGCAGAGGACAGAAACAACA
CL418_F20M13-R	ATGTCATTACGGTTAATACCAAGG
CL501_T32M21-F	GCCAACAGATCCAACGGTTCT
CL501_T32M21-R	TGGATGAGTTCCCTTGAAGAA
CL506_F5024-F	GTGAGGTACTAAGTGAGTTTGACA
CL506_F5024-R	GATACCGACCGGTGAATTTTATAG
CL509_F15A18-F	ATCATCTGCCCATGGTTTTT
CL509_F15A18-R	TTGCTTTTTGGTTATATTCGGA
CL400-F6N15-1F	AGAAAGAACGAGTGGTAGTCC
CL400-F6N15-1R	GTCTTCATCAATTACCCACCTC
CL400-F6N15-2F	GCGGTGAACCGTTACATTTC
CL400-F6N15-2R	ACATCGAACCACACAACTATC
CL400-F5I10-1F	ATCCTCTCGTAACTTTTCCTTC
CL400-F5I10-1R	TCATTCAAGATAACTCCGGCG
CL400-F5I10-2F	TAAGCTATGTTCCCGGACTCC
CL400-F5I10-2R	GTGGAAACATCACACGGATG
CL400-F6N23-F	AGTTCCTTCCATGCCTTAAT
CL400-F6N23-R	CAATCAAATGGAGCCAAATA
CL400-F15P23-F	CTAGCACCTCAGGAGAGTC
CL400-F15P23-R	CATATCCCCGCACGTATG
CL400-T18A10-F	GGTGTCTTGGTCTTGACTAT
CL400-T18A10-R	AAAGCAATGCCAGGGAAC
CL400-F2N1-F	GTCGGACCACAGTTGATAAG
CL400-F2N1-R	ACGGCCCGATTTGTTTAC

CL400-F3D13-F	TGAATAAAAGTTGGTTTATC
CL400-F3D13-R	TCATAATGGCATTTGGTTCA
ACTIN-F	GCCATCCAAGCTGTTCTCTC
ACTIN-R	GCTCGTAGTCAACAGCAACAA
RT-ROS1-F	GATTCTTGTTGATACGCGGGT
RT-ROS1-R	GACATCGTTTCCTCTGAGTCCAA
RT-ROS3-F	TGATCTTGCTGGGCTTCCTAG
RT-ROS3-R	AGTATCTGCAAGTGATCCTGAGTC
RT-IDM1-F	TTGGGTCAGCTTGGAAAGCATTGG
RT-IDM1-R	AAGGCTCTAAGGCACTCCACCAAA
RT-IDM2-F	GTTGATCCGTCTCTAGTCATGAAG
RT-IDM2-R	TTCACCAATGTCCATCAGTCCC
RT-IDM3-F	GAGAAGACGGTTCAAGAACTC
RT-IDM3-R	GCTGAATTCACCGTAATCTTTGC
RT-DME-F	ACCTTTCAAGCTCTGCGTTCA
RT-DME-R	CCTGCCACGAAGGAATTTCA
RT-MBD7-F	GGTTCATCAGAAGAGGAAGCCGC
RT-MBD7-R	GACAGGTCATATATTGAGCCAGGAACAGG
RT-DML3-F	GCAACCAGGTGAAACATCATC
RT-DML3-R	CCTCGCATTGCTGTTCTACAT
RT-DML2-F	GTCCCTTACTTGTTAGCCATTTG
RT-DML2-R	GTGCCATTCAAAGGGAATCC
(q)RT-LUC-F	CGGAAAGACGATGACGGAAA
(q)RT-LUC-R	CGGTACTTCGTCCACAAACA
Chop-region1-F	CCTATGGTCTACTCTCACGCC
Chop-region1-R	TGCTTAAAGCTTGTTGCCAAATCT
Chop-region2-F	ATTTATGACACTGAGATGTGCCCT
Chop-region2-R	CATAGAACTGTACGTGCCTTCATG
Chop-region3-F	TACGTGCCTACTTGCCTCAAC
Chop-region3-R	GGCCCAGTGGGATGAATACAA
Chop-region4-F	GGCAAGGAGATCAAAGTGATGAAG
Chop-region4-R	TTCTTGAACGTTCCGAACGTCTC
Chop-region5-F	TCGTTGTCCCGTACCTCCTAAG
Chop-region5-R	CACCACAGAAAACAGCCCAC
